# Superconductive Coupling Effects in Selectively Grown Topological Insulator-Based Three-Terminal Junctions

**DOI:** 10.1021/acsnano.4c15893

**Published:** 2025-01-13

**Authors:** Gerrit Behner, Abdur Rehman Jalil, Alina Rupp, Hans Lüth, Detlev Grützmacher, Thomas Schäpers

**Affiliations:** † Peter Grünberg Institut (PGI-9), Forschungszentrum Jülich, 52425 Jülich, Germany; ‡ JARA-Fundamentals of Future Information Technology, Jülich-Aachen Research Alliance, Forschungszentrum Jülich and RWTH Aachen University, 52425 Jülich, Germany

**Keywords:** topological insulators, multiterminal Josephson junction, superconductivity, shadow mask, Josephson diode effect, proximity effect

## Abstract

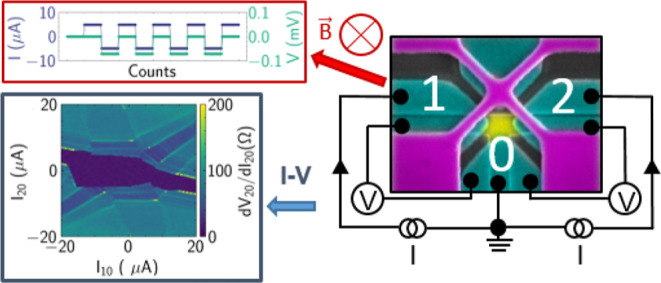

The combination of an ordinary s-type superconductor with three-dimensional topological insulators creates a promising platform for fault-tolerant topological quantum computing circuits based on Majorana braiding. The backbone of the braiding mechanism are three-terminal Josephson junctions. It is crucial to understand the transport in these devices for further use in quantum computing applications. We present low-temperature measurements of topological insulator-based three-terminal Josephson junctions fabricated by a combination of selective-area growth of Bi_0.8_Sb_1.2_Te_3_ and shadow mask evaporation of Nb. This approach allows for the in situ fabrication of Josephson junctions with an exceptional interface quality, important for the study of the proximity-effect. We map out the transport properties of the device as a function of bias currents and prove the coupling of the junctions by the observation of a multiterminal geometry-induced diode effect. We find good agreement of our findings with a resistively and capacitively shunted junction network model.

Three-dimensional topological insulators are a class of materials which recently raised a lot of interest due to its promising applicability in the field of topological quantum computing.^1−3^ The material class exhibits strong spin–orbit coupling. This, in turn, leads to band inversion in the bulk electronic band structure. As a consequence, gapless surface states appear, which are protected by a time-reversal symmetry. Proximizing a topological insulator nanoribbon with an s-type superconductor and aligning a magnetic field along the nanoribbon give rise to Majorana zero modes.^4^ Braiding of these Majorana zero modes is the essential computation operation in topological quantum computing.^5−9^ For this process, multiterminal structures are necessary in which the superconducting phase of the different electrodes needs to be adjusted. The three-terminal Josephson junction therefore represents an important building block for these networks.^10−16^ It is crucial to understand the transport in these devices for further use in topological quantum computing applications. Generally, hybrid devices with multiple connections lead to rich physics in terms of transport properties, with a huge parameter space to be probed.^17^

In recent years, the field of multiterminal Josephson junctions and their unique properties have attracted a lot of interest in the scientific community, e.g., the emergence of *n*-1-dimensional topological properties from an *n*-terminal Josephson junction made from conventional superconductor or the study of the synthetic Andreev band structure in the two-dimensional phase space.^18−22^ While multiterminal Josephson junctions have extensively been studied in epitaxially grown semiconductor-superconductor hybrid structures,^22−26^ not much has been reported on these devices in the field of topological materials. A flux-controlled three-terminal junction based on Bi_2_Te_3_ revealed the opening and closing of a minigap.^27,28^ Furthermore, a three-terminal junction based on the topological insulator Bi_4_Te_3_ was investigated, which did not show the expected signatures of the multiterminal Josephson effect but rather those of a resistively shunted network of two Josephson junctions.^29^

Recently, the superconducting diode effect has attracted a lot of attention.^30^ A characteristic of the diode effect is that the magnitude of the critical supercurrent is dependent on the direction in which the current is driven. The diode effect occurs when both inversion and time-reversal symmetry are broken. For Josephson junctions with a semiconducting^31−34^ or topological insulator^35^ weak link, this can be accomplished by the presence of spin–orbit coupling in conjunction with an external magnetic field for the time-reversal symmetry breaking. Alternatively, the inversion symmetry can be broken by the device layout itself. This can be achieved, for example, by a superconducting quantum interference device, where each of the two junctions of the interferometer has a different current phase relation.^36^ More recently, the asymmetry in a multiterminal Josephson junction has led to a diode effect, either by keeping one of the superconducting electrodes floating^25^ or by phase biasing using superconducting loops connecting pairs of electrodes in the junction.^26^

We present low-temperature measurements of three-terminal Josephson junctions fabricated by a combination of selective-area growth of the topological insulator Bi_0.8_Sb_1.2_Te_3_ and shadow mask evaporation of Nb as the superconductor (see Figure 1).^37,38^ This approach allows for the in situ fabrication of Josephson junctions with very high interface transparency, important for the study of the superconducting proximity-effect. The transport properties of the junction are mapped out as a function of the bias current and magnetic field. The bias current maps show several interesting transport phenomena, e.g., an extended superconducting area and multiple Andreev reflections (MAR), indicating the successful fabrication of a fully coupled three-terminal junction. The measured results for the junction appear to be in good agreement with a resistively and capacitively shunted junction (RCSJ) model. Intrinsic asymmetries of the device and their effect on transport in the junctions are also very well reproduced. The coupling of the junctions is emphasized by the observation of a multiterminal geometry-induced diode effect as a result of an externally applied magnetic field.

**Figure 1:**
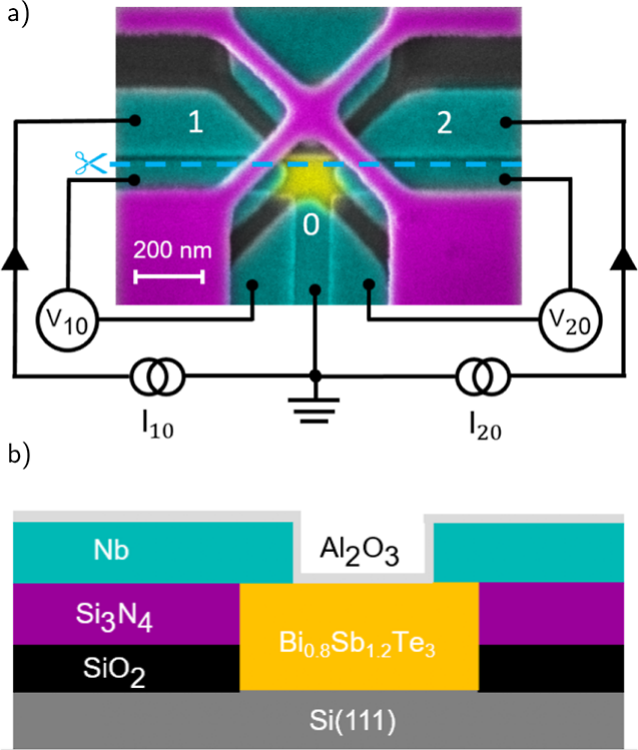
(a) Scanning electron micrograph of the device. The electronic setup used for applying current and measuring voltage in the first three-terminal measurement configuration is also shown. The terminal names (0, 1, 2) that are used for naming the single junctions are indicated in red. The blue dashed line indicates a line cut through the layer stack which is presented in (b). (b) Schematic illustration of the line cut (dashed blue line) in the layer stack making up the device.

## Results and Discussion

### Two-Terminal Characteristics

**Figure 2:**
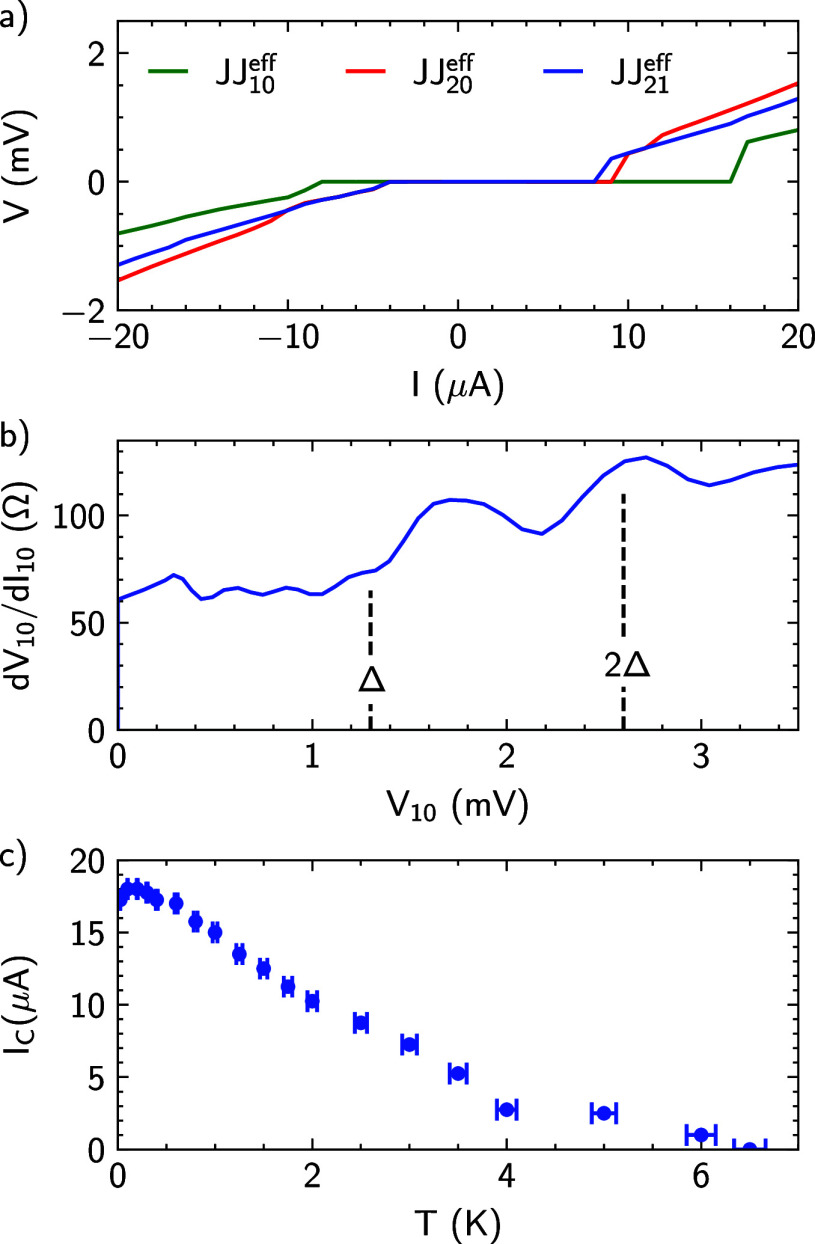
(a) Current–voltage characteristics of the single junctions JJ_10_^eff^, JJ_20_^eff^, and JJ_21_^eff^ in the device shown in Figure 1a. (b) Differential resistance of JJ_10_^eff^ as a function of bias voltage *V*_10_. The position of the voltage bias for possible MAR is indicated by dashed lines, where the first two are labeled 2Δ and Δ. (c) Temperature dependence of the critical current of JJ_10_^eff^.

Here, we discuss the basic characteristics of the effective Josephson junctions that are defined by contacting two of the three terminals, e.g., the bottom and the right superconducting arm. These terminals are indicated in red as 1 and 0 in Figure 1a. Henceforward, the junction will be termed JJ_10_^eff^. The width of the topological insulator ribbons is about 100 nm. The distance between the Nb electrodes is about 70 nm between terminals 1 and 2 and 150 nm between the other pairs of electrodes. Figure 2a shows the DC-characteristics of all three effective junctions, i.e., JJ_10_^eff^, JJ_20_^eff^, and JJ_21_^eff^ at a zero magnetic field. The junctions exhibit a hysteresis between the switching current *I*_c_ and retrapping current *I*_r_. The current–voltage characteristics of Josephson junctions can be described by a RCSJ model. However, because of the coplanar junction geometry and the resulting very low junction capacitance, we can rule out that the hysteresis is due to an overdamped junction characteristics. We rather attribute the hysteresis to Joule heating effects.^39^ Note that JJ_20_^eff^ and JJ_21_^eff^ exhibit similar switching currents slightly below 10 μA, whereas JJ_10_^eff^ shows a critical current larger by almost a factor of 2. We attribute this difference to variations in the interface properties. The switching currents, as well as the other characteristic parameters for all junctions, are summarized in Table 1. In the following, we will discuss the properties of the junction JJ_10_^eff^ as an example.

**Table 1:** Single Junction Parameters: *I*_c_ Critical Current, *R*_N_ Normal State Resistance, andτ Transparency

	JJ_10_^eff^	JJ_20_^eff^	JJ_21_^eff^
*I*_c_	16 μA	9 μA	8 μA
*R*_N_	113 Ω	138 Ω	131 Ω
*I*_exc_	19.1 μA	10.7 μA	12.6 μA
Τ	0.94	0.87	0.89

The excess current *I*_exc_ of the junctions is determined by a linear regression of the junctions’ ohmic behavior at bias voltages larger than 2Δ, with Δ the superconducting gap energy. Here, 2Δ ≈ 2.6 meV is determined from the critical temperature *T*_c_ ≈ 8.5 K of the Nb film. The slope of the linear regression also determines the normal state resistance *R*_N_ of the junction, which is 113 Ω for junction JJ_10_^eff^. The excess current *I*_exc_ ≈ 19.1 μA is used to estimate the junction transparency τ of JJ_10_^eff^. It can be gained by a fit to the analytical calculation of the excess current following the work of Niebler et al. based on the Octavio–Tinkham–Blonder–Klapwijk model.^40−42^ The junction transparency is a figure of merit to evaluate the interface quality. In fact, our junctions exhibit a large transparency, i.e., up to τ = 0.94 for junction JJ_10_^eff^.

Figure 2b shows the differential conductance as a function of bias voltage. Here, features in the differential conductance reveal subharmonic gap structures.^43−48^ The signatures can in most cases be attributed to MAR. They are expected to appear at voltages of *V* = (2Δ)/(*en*), where *e* is the elementary charge and *n* is an integer.^40,49^ The shape of the features is determined by the transport characteristics in the junction.^50−52^ The positions of the voltage biases for possible MAR are indicated by vertical dashed lines, where the first two are labeled 2Δ and Δ. The peak located at roughly 1.7 mV bias voltage *V*_10_ could be a result of the 2Δ MAR resonance due to the induced gap in the TI weak link or in fact a result of a resistance change in one of the other junctions. The exact reason is hard to determine as all junction are evidently coupled to each other, influencing the behavior and measured results. Figure 2c shows the temperature dependence of the switching current of JJ_10_^eff^. The temperature dependence indicates that the behavior of the weak link is dominated by diffusive transport.^37^ We attribute this to the fact that the supercurrent is carried not only by ballistic surface states but also by diffusive bulk states.

### Three-Terminal Measurements

Due to the intrinsic asymmetry of the T-shape of our three-terminal junction, two different three-terminal configurations are probed. The first is schematically shown in Figure 1a. Here, the coupling between the two driving junctions, i.e., from terminals 1 and 2 to terminal 0 at ground, respectively, is mediated by the junction connecting the left and right arm. The characteristics of the three-terminal junction are probed by measuring the differential resistance d*V*_20_/d*I*_20_ between terminals 2 and 0 for the respective configurations. The corresponding bias maps for d*V*_10_/d*I*_10_ are shown in the Supporting Information.

**Figure 3:**
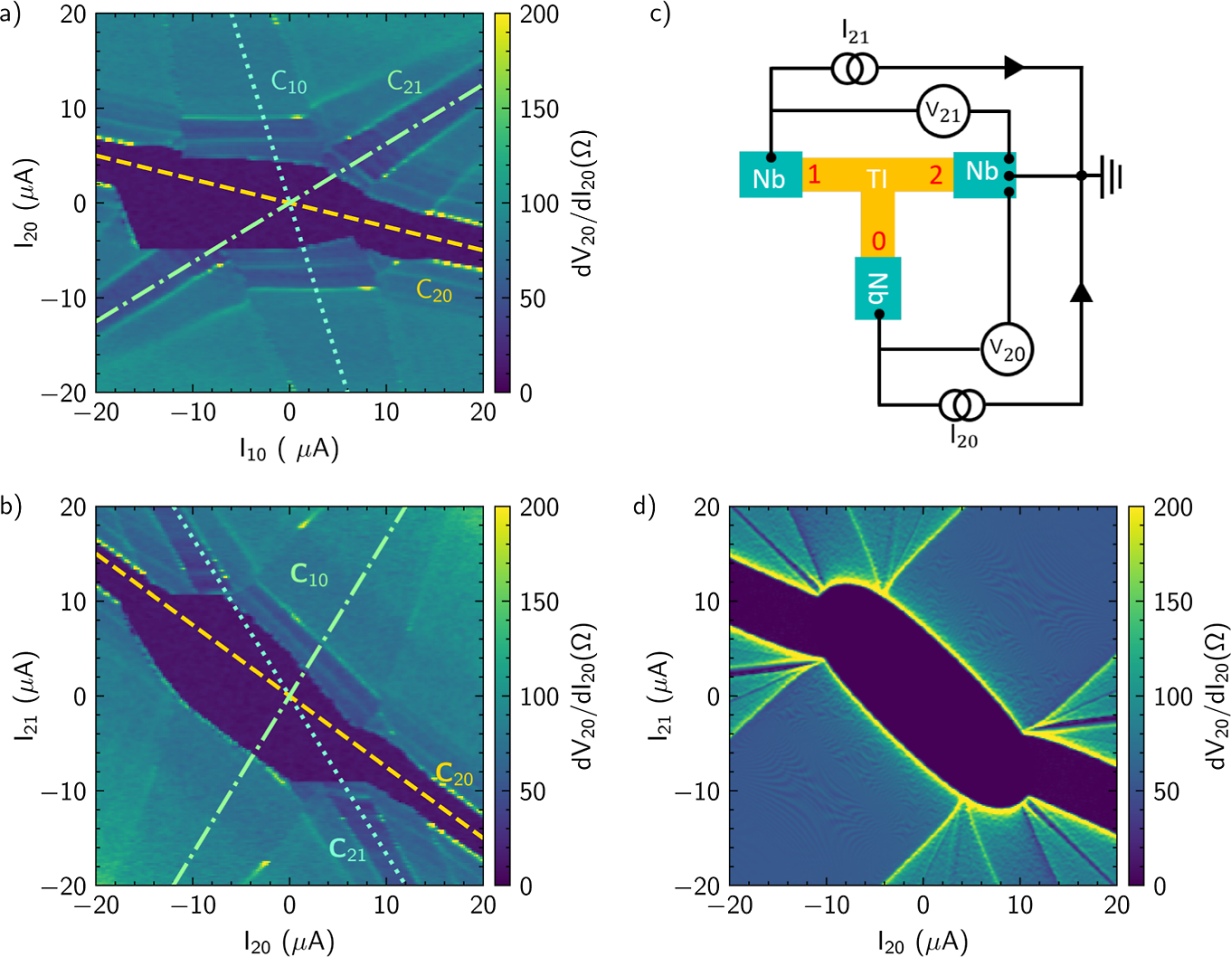
Differential resistance—current bias maps for different measurement configurations. (a) Current bias map of the three-terminal junction measured in the setup shown in Figure 1a. The bias map depicts the differential resistance of d*V*_20_/d*I*_20_ as a function of applied currents *I*_10_ and *I*_20_. The three different lines (dotted, dashed, and dashdot) in the graph indicate the three regimes of compensated currents. The compensated currents are an effect mediated by the nongrounded Josephson junction coupling the two others. In this case, the coupling is mediated by JJ_12_. (b) Current bias map of the measurement configuration shown in (c). The junction determining the coupling between the two grounded junction is switched in order to increase the effects mediated by the coupling. The coupling junction in this case is JJ_20_. (c) Schematic depiction of the electronic setup of the second three-terminal measurement configuration with ground on terminal 2 is also shown. (d) Current bias map generated with the solution of the RCSJ network model. The simulation is carried out with values extracted from the measurements. A direct comparison of the experimental results in (b) and the theoretically expected behavior in (d) gives a reasonable qualitative agreement.

Figure 3a shows the differential resistance d*V*_20_/d*I*_20_ measured in the configuration shown in Figure 1a as a function of applied currents *I*_10_ and *I*_20_. The measurement outcome can be summarized by three prominent features.^17^ The first and most distinct feature is an extended region of superconductivity in the center of the map, indicated by the dark blue area. This corresponds to the zero-voltage state between terminal 2 and 0 of the three-terminal junction in the (*I*_10_, *I*_20_) plane. Since the switching and retrapping current are different, the superconducting area is asymmetric with respect to the center of the current bias map. The second characteristic feature of a three-terminal junction is indicated by three lines in the bias map marked *C*_10_, *C*_20_, and *C*_21_, respectively. They each represent a particular combination of bias currents *I*_10_ and *I*_20_ for which either *V*_10_, *V*_20_, or *V*_21_ is zero (see Figure 3). This is a generic feature of a three-terminal Josephson junction that exhibits dissipationless transport in all of its junctions. They are the result of a current being able to flow to ground by two different paths in the multiterminal junction. For example, current *I*_20_ can not only flow to ground directly via the junction JJ_20_ formed between superconducting electrodes 2 and 0 but also take a detour through the other arms. Thus, part of the current will also flow from terminal 2, via terminal 1, to ground. For example, if *I*_20_ ≥ 0 and *I*_10_ ≤ 0, this will lead to a compensation of currents in junction JJ_20_. As a result, the superconducting region in the bias map is extended along the diagonal. The slope of the extension is determined by the ratio of the normal state resistances of the junctions.^17^ In Figure 3a, line *C*_20_ therefore represents a compensation of currents in the probed junction, whereas line *C*_10_ corresponds to a compensation of currents in junction JJ_10_ formed between electrode 1 and 0, visible as a reduced resistance. Line *C*_21_ represents the compensation in the coupling junction JJ_21_. Here, the condition sgn(*I*_10_) = sgn(*I*_20_) needs to be fulfilled so that the current components provided from terminal 1 and 2 flowing through JJ_12_ have opposite signs.

In order to reveal the effect of the junction properties on the superconducting area, a second configuration shown in Figure 3c is investigated, where now JJ_20_ mediates the coupling between JJ_10_ and JJ_21_. Figure 3b shows the differential resistance d*V*_20_/d*I*_20_ as a function of the applied currents *I*_20_ and *I*_21_. Visible again is an extended superconducting region and the features due to compensating currents indicated by the lines *C*_10_, *C*_20_, and *C*_21_.

**Figure 4:**
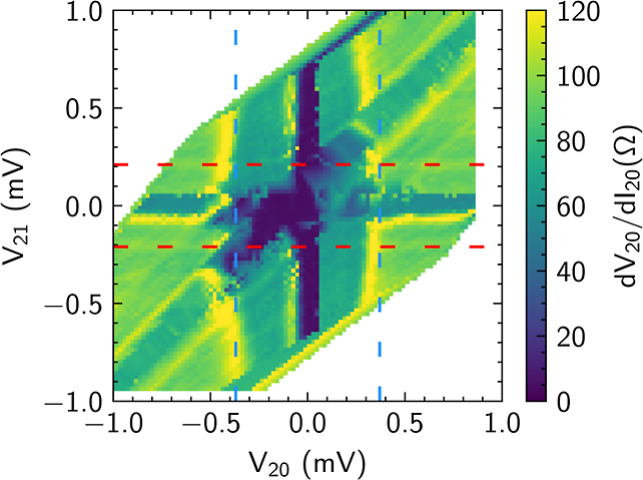
Bias map shown in Figure 3c but now with the differential resistance d*V*_20_/d*I*_20_ plotted as a function of the DC voltage drops *V*_20_ and *V*_21_. The horizontal and vertical lines at zero voltage can be attributed to the superconducting state between the corresponding pairs of terminals. The vertical and horizontal lines marked in blue and red are attributed to MAR in junctions JJ_20_ and JJ_21_, respectively. The diagonal line at *V*_20_ = *V*_21_ corresponds to the superconducting state between the terminals 1 and 0.

The third feature in the current bias maps is equipotential lines, which we attribute to MAR resonances in the junction according to the analysis of Pankratova et al.^17^Figure 4 shows the differential resistance d*V*_20_/d*I*_20_ as a function of the DC voltages *V*_20_ and *V*_21_ recorded in the measurement configuration shown in Figure 3c. The equipotential lines visible in the current bias color maps do indeed appear as lines of constant voltage for the respective junctions. We attribute them to MAR resonances in the sample. However, it is difficult to assign the respective lines to a fixed value of *V* = (2Δ)/(*en*), but it is reasonable to assume that the lines correspond to higher order MAR resonances.

### Simulation

Neglecting multiple couplings, our three-terminal Josephson junction can be described by a model that connects three Josephson junctions, i.e., JJ_10_, JJ_20_, and JJ_21_, in a triangular network. A schematic of the network is provided in the Supporting Information. We employed the simulation approach presented in the work of Gupta et al.^25^ to determine the relevant junction parameters. The *IV* characteristics in the two-terminal measurements presented above are used to extract first estimates of the critical currents and normal state resistances. In the second step, these values are adjusted in a semiqualitative manner until a satisfactory agreement with the experimental data is obtained. The resulting bias map of the simulated three-terminal Josephson junction, corresponding to the measurements shown in Figure 3c, is shown in Figure 3d. An approximate matching of the critical current area as well as the slope and position of the arms is achieved. The corresponding parameters, i.e., critical currents and normal state resistances for each individual junction are presented in the Supporting Information. It turns out that a good matching is obtained by assuming a much higher critical current for junction JJ_10_ compared to the other two junctions. In general, the critical currents of the individual junctions in the network are smaller than the values obtained from the two-terminal measurements because the bypass currents are not included. Since the junction capacitances are negligible and Joule heating is not implemented, the simulation fails to reproduce the hysteresis of the device, which leads to a slight shift from zero in the experimental data. Since in the simulation effects are not included, the experimentally observed tapering of the three arms/slope for the higher current regimes cannot be reproduced. The tapering is a result of the dynamic reduction of the critical current due to Joule heating.

### Diode Effect

**Figure 5:**
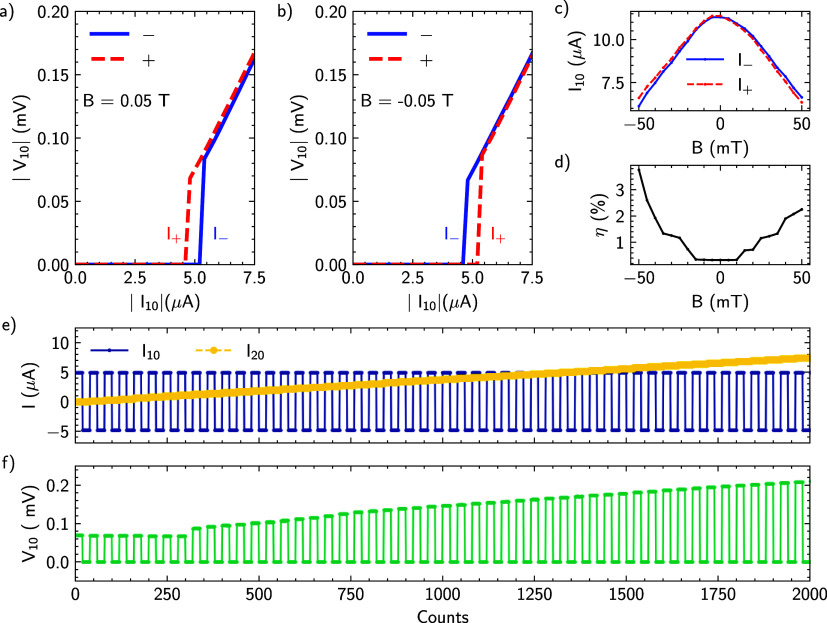
Diode effect in a three-terminal Josephson junction. (a) Current–voltage characteristics of the effective junction JJ_10_^eff^ for negative and positive bias currents at an applied out of plane magnetic field of 50 mT. In this case, the critical current is larger for positive bias currents. (b) Corresponding set of *IV* characteristics after inversing the magnetic field to −50 mT with the critical current for negative bias currents being larger. (c) Switching currents *I*_+_ and *I*_–_ as a function of magnetic field for positive and negative bias currents, respectively. (d) Diode efficiency η as a function of magnetic field. (e) Periodic switching of current *I*_10_ while linearly increasing the control terminal bias *I*_20_. (f) Response of JJ_10_^eff^ to an alternating square-wave current *I*_10_ and a linearly increasing current *I*_20_ shown in (e). A field of 50 mT is applied.

Due to the breaking of inversion symmetry by the device layout, three-terminal Josephson junctions are expected to exhibit a diode effect when exposed to a perpendicular magnetic field.^25,53^Figure 5a shows the current–voltage characteristics of JJ_10_^eff^ with terminal 2 left floating. The current from terminal 1 to terminal 0 was increased from zero in either the positive or negative direction to investigate the switching current for both current directions. By applying a magnetic field of 50 mT, it is found that the switching current for positive bias currents (*I*_+_) is smaller than that for negative currents (*I*_–_). As can be seen in Figure 5b, reversing the magnetic field to −50 mT causes the polarity of the diode effect to switch, with *I*_+_ now being larger than *I*_–_.

In order to quantify the performance of the Josephson diode, its efficiency is defined as follows: η = δ*I*_c_/(*I*_+_ + |*I*_–_|), where δ*I*_c_ = (*I*_+_ – |*I*_–_|). At ±50 mT, we get a diode efficiency η of about 0.04 for JJ_10_^eff^. For a fully symmetric three-terminal Josephson junction, the diode efficiency should follow a Φ_0_/2 periodicity, where Φ_0_ = *h*/(2*e*) is the magnetic flux quantum and *h* is the Planck constant.^25^ A maximum diode efficiency η_max_ of 0.28 is expected at an applied external flux of Φ_0_/4. For our device, the magnitude of the magnetic field corresponding to Φ_0_/4 can be calculated from the junction size estimated from the SEM micrograph shown in Figure 1a. Assuming a junction area of 100 × 100 nm^2^, one obtains a magnetic field of ≈50 mT, corresponding to the field applied in our experiment. The experimentally determined value at ±50 mT of η ≈ 0.04 is considerably smaller than the expected η_max_. We attribute the smaller value to the asymmetric layout of our multiterminal Josephson junctions and to the unbalanced switching currents. Figure 5c displays the critical currents *I*_–_ and *I*_+_ as a function of magnetic field with the evolution of the diode efficiency η shown in Figure 5d. It can be seen that around zero magnetic field both currents *I*_–_ and *I*_+_ are equal yielding η = 0%. When increasing the magnetic field into the respective direction, the difference between *I*_–_ and *I*_+_ becomes larger, yielding increased efficiency. The existence of the superconducting diode effect is a good measure of the quality of the device and can be seen as a proof-of-principle experiment for the quality and coupling of the three-terminal Josephson junction. In our case, the diode effect is effectively a demonstration that our description of the device as a coupling of three Josephson junctions is adequate.

The diode characteristics of the single junction under application of an alternating current are depicted in the Supporting Information. Here, we focus on the characteristics determined by the three-terminal layout. By biasing the second terminal with a current *I*_02_, it is possible to control the diode characteristics.^25^ This is illustrated in Figure 5e,f, where the Josephson bias current *I*_10_ is periodically switched between ±5 μA while the voltage drop *V*_10_ is recorded. At the same time, the control bias current *I*_20_ is ramped from 0 to 15 μA. The voltage drop of JJ_10_^eff^ recorded in Figure 5f shows that at zero control bias current, there is already a diode effect, with a superconducting state at *I*_10_ = −5 μA and a voltage drop of 0.075 mV at +5 μA. As long as the junction JJ_20_ is in the superconducting state, i.e., up to *I*_20_ = 1 μA, the diode characteristics do not change. Beyond this value, however, the voltage drop of the diode makes a sudden jump and increases approximately linearly. For negative diode current biases, the diode remains in the superconducting state up to the maximum control current of 15 μA. Closer inspection reveals that at a current *I*_20_ of about 2.5 μA, the slope of the linear increase in *V*_10_ decreases slightly. We attribute this to the fact that the two other junctions, JJ_20_ and JJ_21_, are in the resistive state at the respective bias current.

## Conclusions

In conclusion, we have fabricated a three-terminal Josephson junction in situ, where the weak link material consists of a selectively grown topological insulator. The superconducting Nb electrodes are defined by shadow evaporation. This approach allows the fabrication of arbitrarily shaped junctions for future quantum computing applications, in particular, structures for braiding Majorana zero modes. The single junctions that make up the device are of very high quality, with high transparency and evidence of MAR. The three-terminal junction exhibits all the characteristics of a fully coupled multiterminal Josephson junction. The observed slight asymmetries in the switching currents are attributed to the intrinsic asymmetry of the T-shaped junction and to variations in the interface transparency. The bias current maps of the differential resistance show all features expected for multiterminal Josephson junctions where all junctions interact in the superconducting state. The proof of principle now allows for more sophisticated experiments to detect signatures of the topological superconductivity. By leaving one terminal floating and applying an external magnetic field, a diode effect is observed, where the switching current depends on the direction of the bias current. As a next step, connecting superconducting electrodes by a superconducting loop to achieve phase bias would allow for an efficient control of the diode characteristics, offering a great potential for superconducting electronic circuits.^26^

## Methods

### Fabrication

The samples are fabricated using a combination of selective-area growth and shadow mask evaporation.^37,38^ This gives the possibility to prepare samples with arbitrary geometry and exceptional interface transparency between the topological insulator and the parent superconductor. 10 nm of SiO_2_ and 25 nm of Si_3_N_4_ are deposited by thermal oxidation and plasma-enhanced chemical vapor deposition (PECVD), respectively, on a Si(111) wafer. Trenches in the shape of a T and a width of 100 nm are etched into the stack by means of a resist process using electron beam lithography and reactive-ion etching (RIE). A second stack of 300 nm SiO_2_ and 100 nm Si_3_N_4_ is deposited using PECVD. This stack is subsequently used to define the bridge for shadow evaporation. To do so, the second Si_3_N_4_ layer is patterned into the shape of the bridge using a negative resist process and RIE. By etching the sample with hydrofluoric acid in the second etching step, the bridge is under-etched creating a suspended shadow mask above the trench. The TI growth takes place under rotation of the sample around its normal axis. This ensures homogeneous growth under the shadow mask. The Nb contacts are deposited in situ. For this purpose, 50 nm of Nb is deposited from an angle without rotation of the sample. The shadow mask then patterns the Josephson junction itself, without the need for etching. Finally, the sample is capped by using a 5 nm layer of Al_2_O_3_ to prevent oxidation. The coarse shapes of the electrodes are defined ex situ using an SF_6_ RIE process without damaging the junction area or the nanoribbon at any point.

### Measurements

The sample characteristics were measured in a dilution refrigerator with a base temperature of *T* ≈ 10 mK. Figure 1a shows a typical measurements configuration, where two current sources supply currents *I*_10_ and *I*_20_ from the left and right terminal, i.e., terminals 1 and 2, respectively, to the bottom electrode, i.e., terminal 0, respectively. Voltages *V*_10_ and *V*_20_ are measured accordingly. The voltages are measured in a quasi-four-point measurement scheme. The differential resistance of the sample is measured using a lock-in amplifier by the addition of a 10 nA AC current to the applied DC current. For the measurements of the diode effect, an out-of-plane magnetic field is applied.

## Supporting Information Available

Corresponding counterparts of the bias maps, simulation and differential resistances for all of the junction in the three-terminal junctions; and behavior of the device under application of an magnetic field and an extension of the diode characteristic measurements (PDF)
