# Supplementary material for: Superconductive Coupling Effects in Selectively Grown Topological Insulator-Based Three-Terminal Junctions
Source: ACS Nano. 2025 Jan 13;19(3):3878–85. doi: 10.1021/acsnano.4c15893 (PMC11781021; doi:10.1021/acsnano.4c15893)
Supplement: Supplementary file 1 — nn4c15893_si_001.pdf [file nn4c15893_si_001.pdf]

Supplemental Material: Superconductive coupling effects in selectively-grown topological insulator based three-terminal junctions

Gerrit Behner[1,2,*] Abdur Rehman Jalil[1,2] Alina Rupp[1,2]
Hans Lüth[1,2] Detlev Grützmacher[1,2] and Thomas Schäpers[1,2,†]

[1] *Peter Grünberg Institut (PGI-9), Forschungszentrum Jülich, 52425 Jülich, Germany*
[2] *JARA-Fundamentals of Future Information Technology, Jülich-Aachen Research Alliance, Forschungszentrum Jülich and RWTH Aachen University, 52425 Jülich, Germany*

(Dated: December 29, 2024)

SUPPLEMENTAL NOTE 1: BIAS MAPS

Here, we present the corresponding counterparts to all current or voltage bias maps shown in the main text. Supplemental Figure 1 a) shows the differential resistance dV_{10}/dI_{10} measured between terminals 1 and 0 as a function of applied currents I_{10} and I_{20}. In contrast to Figure 3 a) in the main text, the superconducting region extends along C_{10} corresponding to the compensation of currents in junction JJ_{10}. The superconducting region of JJ_{20} appears as a region of reduced resistance along C_{20}, while the compensation of currents in JJ_{21} shows up as a region of reduced resistance along C_{21}. The same is true for Supplemental Figure 1 b), which shows the corresponding counterpart to the map in Figure 3 b) given in the main text with the differential resistance dV_{21}/dI_{21} plotted as a function of the applied currents I_{10} and I_{21}. Here, the superconducting region extends along C_{21} corresponding to the compensation of currents in the junction JJ_{21}. The compensating currents leading to supercurrents in junctions JJ_{20} and JJ_{10} appear as a region of reduced resistance along C_{20} and C_{10}, respectively. Supplemental Figure 1 c) shows the differential resistance dV_{21}/dI_{21} as a function of the DC voltages V_{20} and V_{21}. Here, the region of zero resistance extends along the horizontal line corresponding to $V_{21} = 0$. Similar to Figure 4 in the main text, the lines of constant voltage due to multiple Andreev reflections are marked with horizontal and vertical dashed blue lines, respectively. Supplemental Figure 1 d) shows the corresponding counterpart to the simulation data for the experimental data given in Supplemental Figure 1 b).

SUPPLEMENTAL NOTE 2: SIMULATION

This section provides details about the simulation and the corresponding parameters presented in the main section. Supplemental Figure 2 depicts the network of coupled resistively and capacitively shunted junctions. The three terminals are marked 0, 1, and 2. The corresponding junctions and the parameters are marked JJ_{10}, JJ_{20}, and JJ_{21}, respectively. A summary of the parameters used is given in Supplemental Table I. Both critical current $\mathrm{I_c}$ and the normal state resistances $\mathrm{R_N}$ are chosen smaller than the measured values. This is necessary to reproduce the features of the measurement, since the measured parameters are only the effective values and a consequence of the effective junction model, e.g. $\mathrm{JJ}_{10}^{\mathrm{eff}}$, explained in the main section. The capacitance for each junction is set to 5 fF. As shown by Graziano et al. [1], a small capacitance leads to an elliptical shape of the superconducting region, as observed in our experiments. For our junction layout, a small capacitance is expected because the shape of the junction represents a coplanar rather than a plate capacitor, as in the case of classical superconductor-insulator-superconductor (e.g., $\mathrm{Al/AlO}_x\mathrm{/Al}$) junctions. The capacitance of the junctions can be estimated by extracting the McCumber parameter β_C as follows:

$$\frac{I_\mathrm{c}}{I_\mathrm{r}} \approx \frac{4}{\pi} \cdot \frac{1}{\sqrt{\beta_C}} \tag{S1}$$

,

$$\beta_C = \frac{2\pi}{\Phi_0} I_C R^2 C. \tag{S2}$$

* g.behner@fz-juelich.de
† th.schaepers@fz-juelich.de

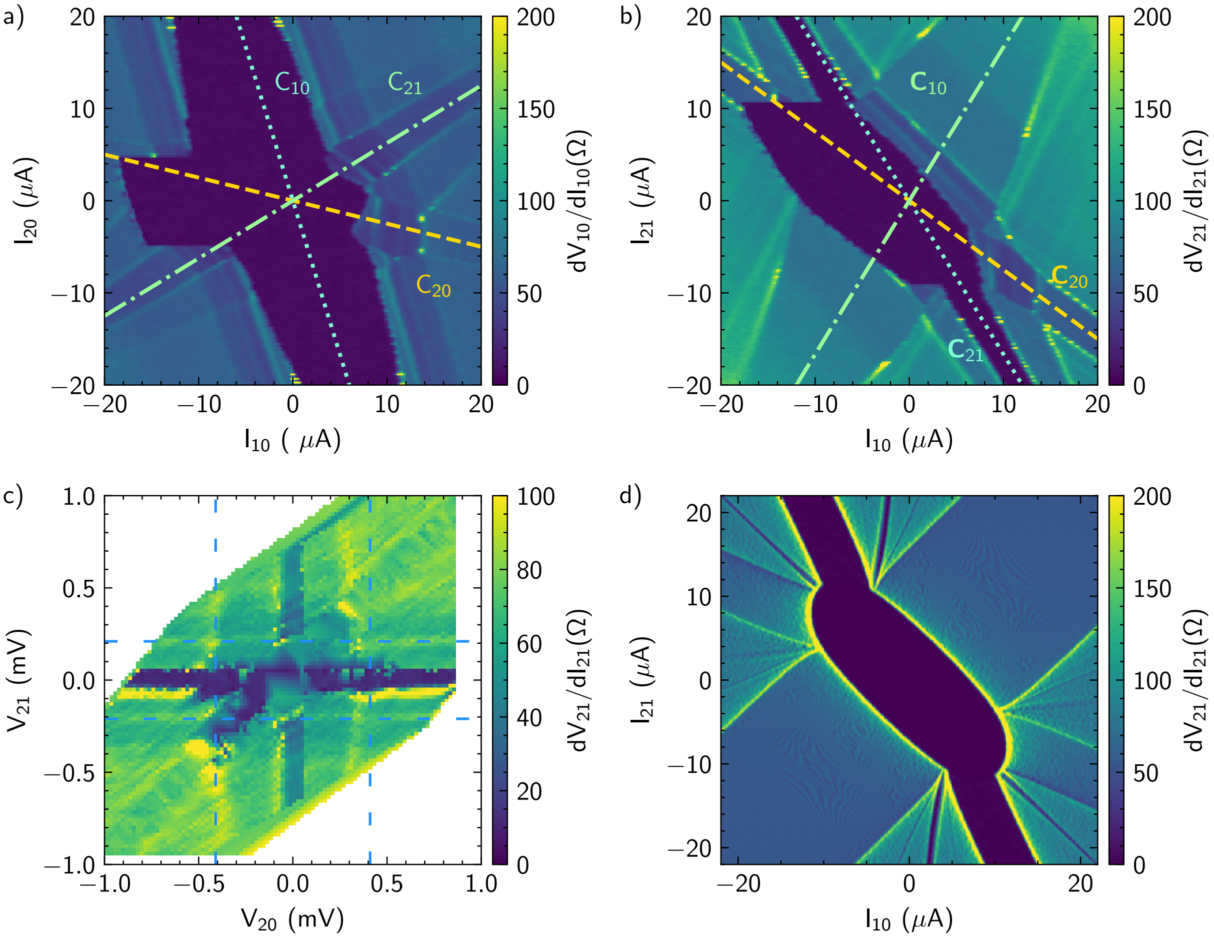

Supplemental Figure 1. **a)** Differential resistance of dV_{10}/dI_{10} as a function of applied currents I_{10} and I_{20} measured in the setup shown in Figure 1 a) in the main text. The three different lines C_{10}, C_{20}, and C_{21} indicate the three regimes of compensated currents. **b)** Current bias map of dV_{21}/dI_{12} for the measurement configuration show in Figure 3 c) in the main text. **c)** Differential resistance dV_{21}/dI_{21} shown in b) now plotted as a function of the DC voltage drops V_{20} and V_{21}. **d)** Current bias map generated with the solution of the RCSJ network model corresponding to the measurements shown in b).

These considerations yield estimation for the capacitances of $(\mathrm{C}_{10}^{\mathrm{eff}}, \mathrm{C}_{20}^{\mathrm{eff}}, \mathrm{C}_{21}^{\mathrm{eff}}) = (3.9\,\mathrm{fF}, 5.9\,\mathrm{fF}, 5.8\,\mathrm{fF})$, proving 5 fF to be a reasonable choice for the capacitance used in the simulation. The same is true, when calculating the co-planar capacity of our device layout in a classical way. A more detailed look on the simulation approach used in this paper can be found in the supplementary material of reference [2].

	JJ_{10}	JJ_{20}	JJ_{21}
I_c	8 μA	4 μA	3.5μA
R_N	100 Ω	105 Ω	105 Ω
C	5 fF	5 fF	5 fF

Supplemental Table I. RCSJ simulation parameters: I_c critical current, R_N normal state resistance, C capacitance.

SUPPLEMENTAL NOTE 3: MAGNETIC FIELD DEPENDENCE

In this section we present the effect of an perpendicular magnetic field on the transport in the junctions. Supplemental Figures 3 a) and b) show the bias map corresponding to Supplemental Figure 1 a) and Figure 3 a) in the main text, respectively, when a magnetic field of 50 mT is applied. As expected, the junction properties decrease with the

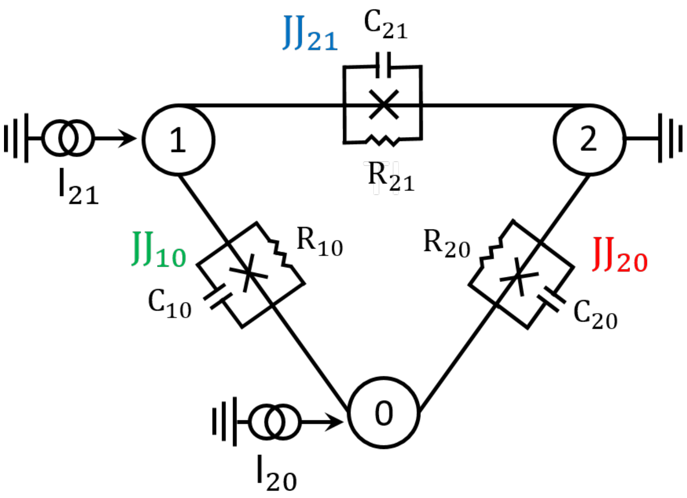

Supplemental Figure 2. Effective transport model used for the simulations shown in the main section. The model is based on a network of three JJs described by a coupled resistively and capacitively shunted junction models The respective parameters are provided in Table I.

application of the field. The features that characterize a multi-terminal junction are strongly suppressed. One finds that the central regions of the Josephson supercurrent are shrunk, with the individual critical current reduced by more than a factor of two. The diagonal features resulting from compensating currents in the junctions, characterized by the lines C_{10}, C_{20} and C_{21}, are almost vanished from the bias map. The lines attributed to the presence of multiple Andreev reflections are barely visible.

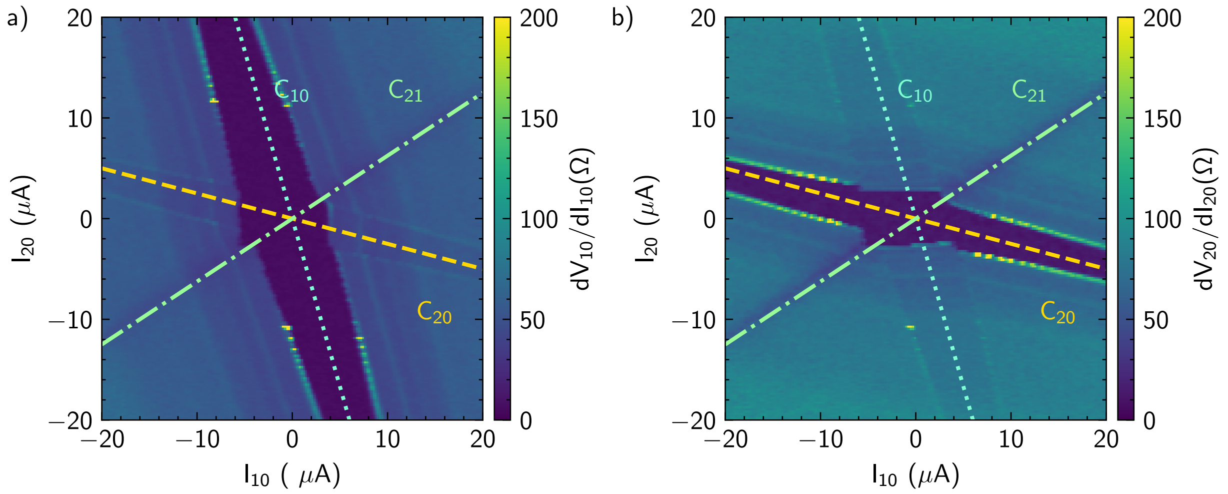

Supplemental Figure 3. **a)** Differential resistance dV_{10}/dI_{10} as a function of currents I_{10} and I_{20} under application of an perpendicular magnetic field of 50 mT. The diagonal lines C_{10}, C_{20}, and C_{21} indicate the features due to compensation currents in the junctions. **b)** Corresponding current bias map with dV_{20}/dI_{20} plotted instead of dV_{10}/dI_{10}.

Supplemental Figure 4 shows the differential resistance dV_{10}/dI_{10} as a function of an perpendicular magnetic field and bias current I_{10}. The map shown in Figure 4 a) is taken starting from 0 mT and sweeping up to the maximum field of -500/500 mT, while recording individual IV curves at the respective field points. The dark blue regions represent the superconducting state. While the map shows signs of periodic behavior for positive fields, corresponding to a Fraunhofer interference pattern, the negative side shows a completely different asymmetric behavior. Judging from the size of the device extracted from the scanning electron microscopy image shown in Figure 1 a) in the main text, the expected magnetic field periodicity should be in the order of $250 - 300$ mT. The local minimum of the critical current found at about 300 mT is in the expected range. We attribute the strongly suppressed supercurrent on the negative magnetic field side to a trapped vortex. With Nb as a type II superconductor, the device is susceptible to

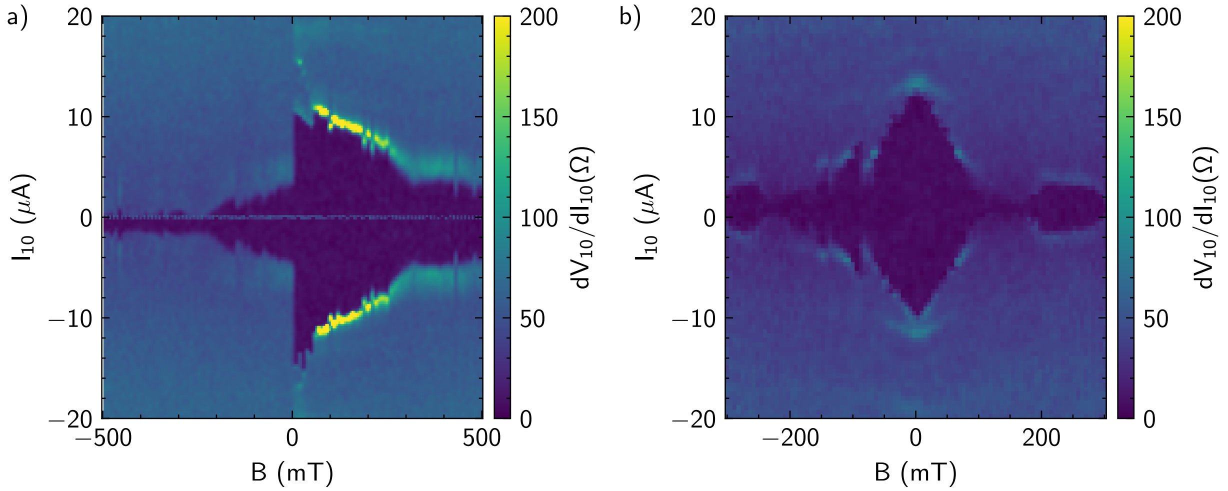

Supplemental Figure 4. Differential resistance dV_{10}/dI_{10} of JJ_{10} as a function of an perpendicular magnetic field B and current dI_{10}, recorded in two different ways. **a)** The magnetic field is swaped from zero in the respective direction while measuring the current voltage characteristics at every individual magnetic field point. **b)** The differential resistance dV_{10}/dI_{10} is recorded as a function of bias current for a magnetic field starting from $-300\,$mT and then consecutively increased to $300\,$mT.

flux trapping when an perpendicular magnetic field is applied. This trapped flux exposes the junction to an effective magnetic field, resulting in an abrupt change in junction behavior.

In the Supplemental Figure 4 b) dV_{10}/dI_{10} is plotted starting at $-300\,$mT and then successively sweeping up to $300\,$mT, recording individual IV curves at the respective field points. The behavior of the junction appears to be completely different, with a central lobe of superconductivity extending to about $-100/100\,$mT, but drifting to an asymmetric behavior for higher fields. We attribute this to two effects, i.e. a non-uniform current distribution at the junctions and the flux trapping that occurs at large fields.

SUPPLEMENTAL NOTE 4: DIODE EFFECT

Supplemental Figure 5 b) shows the response of the junction JJ_{10}^{eff} to an applied square-wave pulse at an applied magnetic field of $50\,$mT. It demonstrates the rectification of a current signal with amplitude $\mathcal{A} = 5\,\mu$A. The amplitude $\mathcal{A}$ of the square wave is chosen so that $|I_-| < \mathcal{A} < I_+$. For negative currents the device remains superconducting, i.e. $V = 0\,$mV, while for positive currents the device becomes resistive with a voltage drop of $V_{10} \approx 70\,\mu$V. No punch-through errors are observed over tens of cycles. This demonstrates the stability of the effect even though the diode efficiency is rather low.

Supplemental Figure 5 c) and d) are the corresponding counterparts of a) and b) at an applied field of $-50\,$mT. As described in the main section on the diode effect, the polarity of the effect should be reversed when the magnetic field is reversed. This is demonstrated here by inverting the rectification of a current signal with amplitude $\mathcal{A} = 5\,\mu$A. In contrast to the measurements shown in Supplemental Figure 5 b), the device remains superconducting for positive currents, i.e. $V = 0\,$mV, while it becomes resistive for negative currents with a voltage drop of $V \approx -70\,\mu$V. Similarly, no punch-through errors are observed over the entire measurement cycle.

Supplemental Figure 6 a) and b) show the complete measurement of the rectification of a current signal of amplitude $\mathcal{A} = 5\,\mu$A applied from terminal 1 to 0 while the second terminal is biased with a current I_{20}. The measurement is taken at a magnetic field of $50\,$mT. The second terminal bias current I_{20} is ramped up to a total current of $15\,\mu$A while I_{10} is periodically switched between $\pm 5\,\mu$A. For negative currents the device remains superconducting, i.e. $V = 0\,$mV, while for positive currents the device becomes resistive. The slight bends at a second terminal bias of about $1\,\mu$A and $2.5\,\mu$A correspond to the junctions JJ_{20} and JJ_{21}, which become resistive. For I_{20} larger than about $3\,\mu$A, a linear increase of the voltage V_{10} with increasing I_{20} is observed. The key features of the measurement are discussed in the main text. No punch-through errors are recorded throughout the measurement.

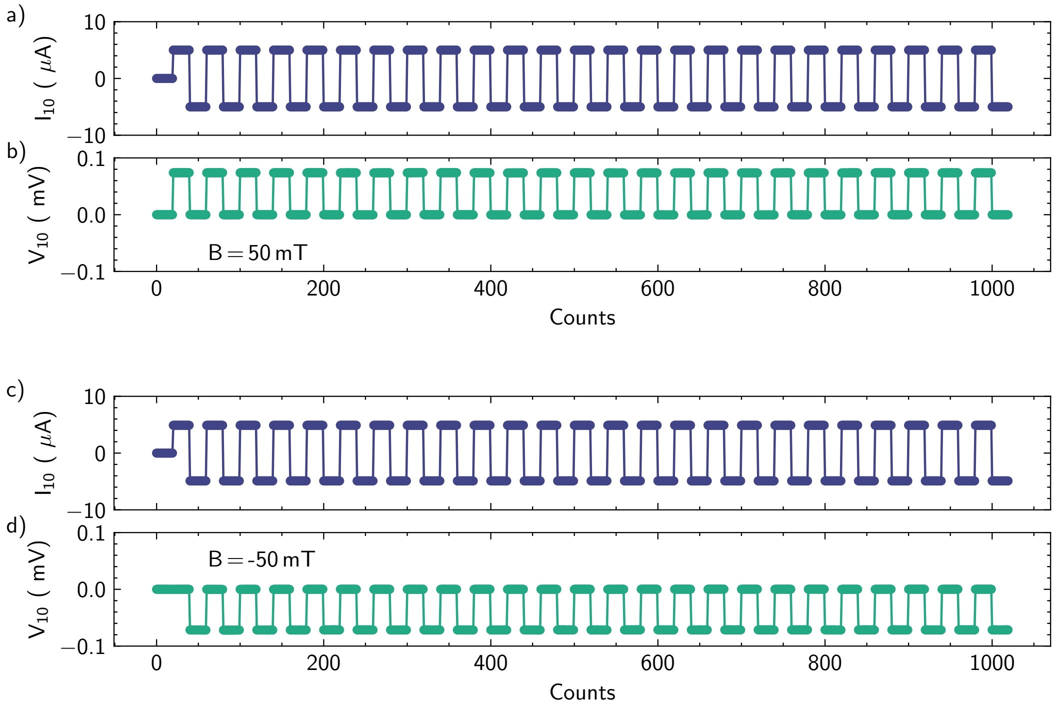

Supplemental Figure 5. Demonstration of rectification of a current signal with amplitude $\mathcal{A} = 5\,\mu$A in an perpendicular magnetic field. **a)** Applied square wave bias current signal with amplitude $\mathcal{A} = 5\,\mu$A. **b)** Corresponding voltage drop V_{10} at the junction $\mathrm{JJ}_{10}^{\mathrm{eff}}$ for a magnetic field of 50 mT. **c) d)** Square wave bias current with amplitude $A = 5\,\mu$A and corresponding voltage drop V_{10} for a magnetic field of -50 mT.

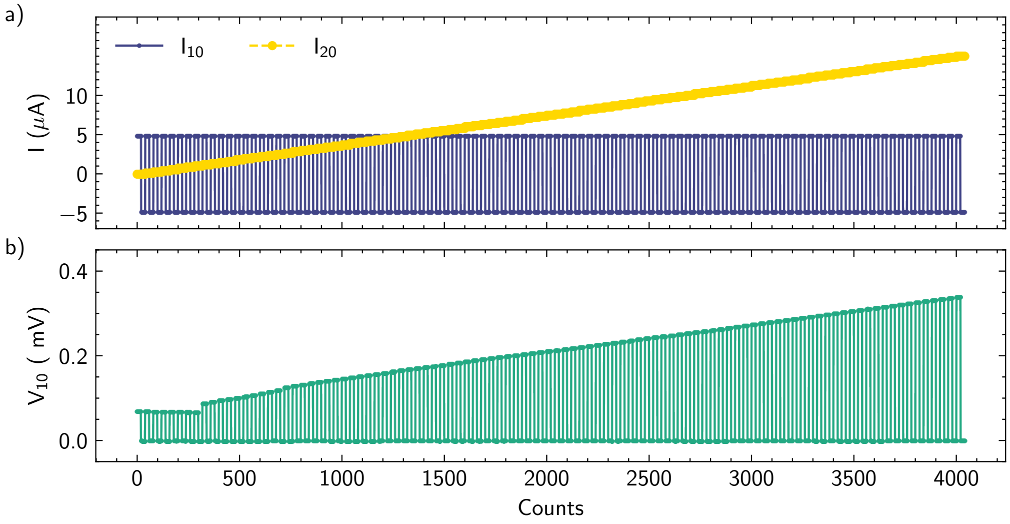

Supplemental Figure 6. Rectification of a current signal in $\mathrm{JJ}_{10}^{\mathrm{eff}}$ while biasing the second terminal with a current I_{20} at an applied field of 50 mT. **a)** Square wave signal with an amplitude $\mathcal{A} = 5\,\mu$A in blue and the linearly increasing second terminal bias current I_{20} in yellow. **b)** Voltage response V_{01} of junction $\mathrm{JJ}_{10}^{\mathrm{eff}}$ to the applied signal shown in a).

SUPPLEMENTAL NOTE 5: DIFFERENTIAL IV-CURVE

Supplemental Figure 7 a) and b) shows the differential resistance of $\mathrm{JJ}_{20}^{\mathrm{eff}}$ and $\mathrm{JJ}_{21}^{\mathrm{eff}}$ as a function of the DC voltages V_{20} and V_{21}, respectively. Similar to Figure 1 b) in the main text, the positions of possible multiple Andreev reflections are marked with vertical dashed lines labeled with 2Δ and Δ, respectively.

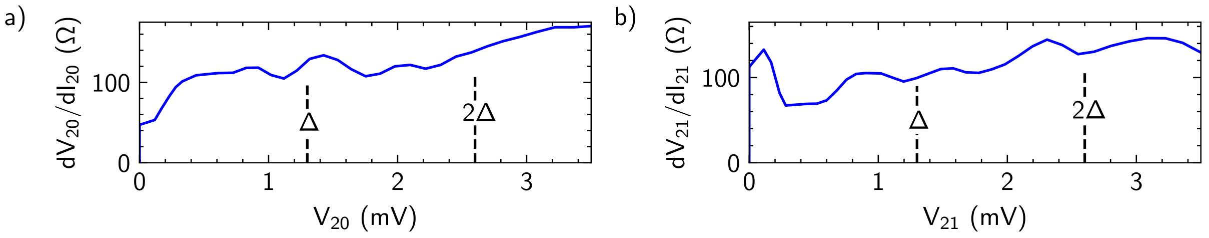

Supplemental Figure 7. **a)** and **b)** Differential resistances of the junctions JJ_{20}^{eff} and JJ_{21}^{eff} as a function of the DC voltages V_{20} and V_{21}. The positions of possible MAR are marked with 2Δ and Δ, respectively.

SUPPLEMENTAL REFERENCES

[1] G. V. Graziano, J. S. Lee, M. Pendharkar, C. J. Palmström, and V. S. Pribiag, Physical Review B **101**, 10.1103/physrevb.101.054510 (2020).

[2] G. V. Graziano, M. Gupta, M. Pendharkar, J. T. Dong, C. P. Dempsey, C. Palmström, and V. S. Pribiag, Nature Communications **13**, 10.1038/s41467-022-33682-2 (2022).
